# Conceptual Anchors in Longitudinal Qualitative Health Research: Using a Methodological Adjunct in Longitudinal Interpretative Phenomenological Analysis

**DOI:** 10.1111/jan.70355

**Published:** 2025-11-04

**Authors:** Kelda J. Folliard, Kenda Crozier, Meghana M. Wadnerkar Kamble

**Affiliations:** ^1^ Maternity Department Norfolk and Norwich University Hospital Norwich UK; ^2^ School of Health Sciences University of East Anglia Norwich UK; ^3^ School of Nursing and Midwifery Queens University Belfast Belfast UK

**Keywords:** health research, interpretative phenomenological analysis, longitudinal qualitative research, midwife research, nurse research, phenomenology

## Abstract

**Aims:**

To highlight how Longitudinal Experiential Concepts can be used as conceptual anchors within Longitudinal Interpretative Phenomenological Analysis to gain temporal interpretative phenomenological insights, a lack of which can be a criticism levelled at novice nurse or midwife researchers utilising phenomenological research methods.

**Design:**

Longitudinal Experiential Concepts were utilised as a novel methodological adjunct to Longitudinal Interpretative Phenomenological Analysis in a study of the lived experience of perinatal anxiety by a midwife researcher.

**Method:**

Longitudinal Experiential Concepts were identified following assimilation of Group Experiential Themes and while building the interpretative narrative account across all three data collection time points, with reflexive annotations facilitating their formulation.

**Results:**

Within a longitudinal vertical (by time point) analysis, Longitudinal Experiential Concepts can add a horizontal view, giving a contemporaneous and dynamic perspective on the experiential threads woven throughout the temporal experience. Use of these conceptual anchors, enabled with reflexive prompts, can prevent the fragmentation that potentially occurs when examining moments in time in Longitudinal Qualitative Research, facilitate clarity in the temporal view of the whole phenomenon and enable phenomenological insights.

**Conclusion:**

A novel addition to the Longitudinal Interpretative Phenomenological Analysis method, Longitudinal Experiential Concepts as conceptual anchors can encourage deeper holistic thinking about the less immediately obvious facets of experience and temporal progression and give the novice nurse or midwife researcher a means to robustly access the phenomenological attitude. These principles may be applicable more broadly within other Longitudinal Qualitative Research approaches.

**Implications for the Profession:**

The use of Longitudinal Experiential Concepts in Longitudinal Interpretative Phenomenological Analysis can enable nurses, midwives, and other clinical health researchers to produce high‐quality, robust longitudinal phenomenological research. This is important due to the popular use and value of these methods aiming to generate new understanding of health conditions and improve patient care.

**Patient or Public Involvement:**

Patients and members of the public were involved in the design of the original research study. Their contributions included reviewing study plans, ensuring the research was in line with the priorities of women experiencing poor perinatal mental health, guiding the researchers on the acceptability of the proposed approach to recruitment and data collection and reviewing participant information and study marketing materials. We gratefully acknowledge Get Me Out the Four Walls, Norfolk, for their support enabling this.

**Summary**

- Impact
  ○ This paper assists with the practical application of Longitudinal Interpretive Phenomenological Analysis, supporting nurse and midwife researchers to overcome methodological and interpretative challenges and in doing so address some of the criticism of nursing phenomenological research.
  ○ Longitudinal Experiential Concepts can help overcome the fragmentation that potentially occurs when examining moments in time in Longitudinal Qualitative Research and facilitate interpretation of the temporal view of the whole phenomenon, enabling rigorous phenomenological insights.
  ○ This methodological adjunct will impact any nurse, midwife or other health researcher wishing to robustly generate new knowledge using Longitudinal Interpretative Phenomenological Analysis.
- What does this paper contribute to the wider global community?
  ○ The findings are applicable to researchers using Longitudinal Interpretative Phenomenological Analysis globally and in any setting.
  ○ The findings may be applicable for use with other Longitudinal Qualitative Research methods.

## 1 Introduction

Clinical experience is commonly the basis for research questions in nursing and midwifery, arising from a desire to generate inquiry and improve patient care (Adams 2025). The first author's decision to examine the lived experience of perinatal anxiety arose from providing midwifery care for women experiencing anxiety and recognising unanswered questions about the nature of the condition from the individual woman's perspective (Folliard et al. 2020). Exploration of living with a health condition and the meaning attributed to individual experiences can be approached via a multitude of qualitative methods, or through surveys or observational studies and grounded in varied ontological principles, one of which is phenomenology.

Longitudinal Interpretative Phenomenological Analysis (LIPA) was the methodological choice for the authors' examination of perinatal anxiety, due to enabling holistic exploration of the meaning individuals derive from the embodied, cognitive affective and existential processes of anxiety across the continuum of pregnancy and post‐birth (Smith et al. 2021). The aim of this was to inform recommendations for clinical midwifery practice. However, a pragmatic and epistemological challenge arose during analysis. This concerned the ability to maintain a sense of temporal progression and idiographic focus true to interpretative phenomenological enquiry, as a clinically active midwife researcher juggling competing demands and aware of the critique of nurse researchers producing high‐quality phenomenological qualitative research (Paley 2016).

This paper discusses the origin, application and value of Longitudinal Experiential Concepts (LECs) as a methodological adjunct within LIPA, contextualised within the challenges clinically active nurses and midwives can face in conducting longitudinal qualitative research. Background to the Interpretative Phenomenological Analysis (IPA) and LIPA methods is provided, with detail on the source perinatal anxiety data and interpretative issues that became apparent. The findings demonstrate how LECs were derived iteratively through the interpretative process, with visual examples and reflexive perspectives provided for illustration. Guidance on the use of the method is provided to enable replication.

## 2 Background

IPA has been used widely in nursing research, due to its utility in examining perspectives on chronic and long‐term illness (McInally and Gray‐Brunton 2021). Longitudinal qualitative research is an emerging methodology in the nursing field, exploring the evolution of experience over time including key transitional and time points within health and illness, but with limited nursing research literature guiding its use (Tuthill et al. 2020). The investigation of lived experience should illuminate the subjective understanding of living through a situation and may be in response to evolving events (Neale and Holland 2012). LIPA aims to capture temporality and can be useful in understanding the changes in experience and perception when researching an event such as becoming a parent (Smith et al. 2021), with the perinatal period a time of significant life change.

**FIGURE 1:**
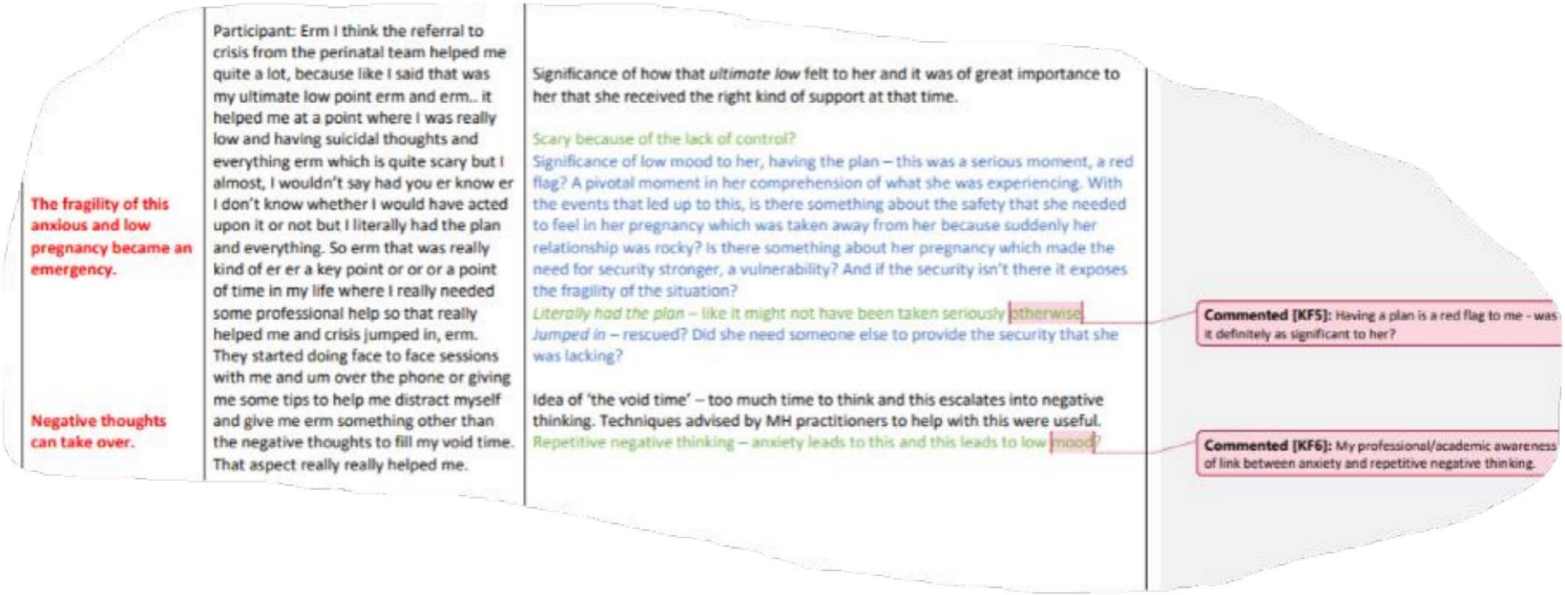
Example of source data with evolving interpretations, reflexive annotations and the emergence of Personal Experiential Statements.

IPA involves a series of methodological steps which are considered common processes rather than a prescribed set of instructions (Smith et al. 2021). The researcher initially revisits each interview transcript on multiple occasions, and through their reflexive annotations begins to build a picture of the meaning within the participant's account and language (Figure 1). The annotations are synthesised into statements representing individual experiences; these are then grouped to identify themes for each participant. Once these Personal Experiential Themes (PETs) have been identified, the interview transcripts are revisited to locate excerpts that evidence the themes. At this stage of case analysis, the researcher has a sense of individual experience. To enable cross‐case analysis, and explore shared and divergent experience, the PETs are further synthesised to produce Group Experiential Themes (GETs), representing experiences across all participants. Finally, a narrative interpretative thematic analytical account is produced which validates the development of the Group Experiential Themes evidenced by interview excerpts.

Longitudinal IPA (LIPA) is a more complex design, gaining attention with an increasing body of literature describing this approach and highlighting its challenges and opportunities (Wanat et al. 2025). A review by Farr and Nizza (2019) of longitudinal designs using IPA indicated that this design can deepen understanding of the dynamic evolution of a phenomenon, allowing fuller comprehension of change. Indeed, longitudinal qualitative research is situated within the interpretivist tradition grounded in hermeneutic and phenomenological schools of thought (Neale 2021). Longitudinal qualitative studies in healthcare can also be valuable for understanding how care provision which changes over time may influence patient experience (Calman et al. 2013). This is relevant for a perinatal study, conducted over a period during which health professional input is intense during pregnancy and less so postnatally, and reflective of other scenarios in which the frequency of healthcare service contact varies over the course of illness.

In LIPA, two approaches to temporal analysis have been identified among published studies, analysis by time‐point (vertical) or by themes spanning time (horizontal) (Farr and Nizza 2019). Although both approaches to LIPA can provide insights into temporal progression, the researcher has a choice, either to complete analysis for a specific time point before moving on to the next (time‐point analysis) or wait until data collection for all time points is complete before analysing the data in its entirety to identify themes (themes spanning time). With the vertical approach, reading the collection of time‐point specific themes and considering how these contribute to progression illuminates the temporal process (Farr and Nizza 2019). Processual understanding in longitudinal qualitative research, the ways in which chronologies are constructed and different aspects of the process of experience connected, must be addressed, and can be enabled using processual questions to interrogate the data (Neale 2021). The need to account for temporal progression is the key difference between IPA and LIPA and so should be carefully considered.

In the perinatal anxiety study described here, a vertical (by time point) analytical choice was made for pragmatic reasons. The Longitudinal Experiential Concepts (LECs) provided a thread through the participant experience, connecting temporal points and giving rise to the ‘conceptual anchor’ notion of this methodological adjunct. This facilitated longitudinal analysis as these conceptual anchors guided the interpretation, allowing a point of reference across time points which could be revisited to illuminate the interconnected characteristics of anxiety over the evolution of experience. The use of the LECs as conceptual anchors also enabled a level of interpretation that provided the idiographic detail required for robust use of the LIPA method (Smith et al. 2021). This was in part by the LECs acting as a vehicle for deeper exploration of elements of experience that were nuanced and phenomenological, requiring careful examination to uncover meaning.

## 3 Data Sources

The perinatal anxiety study obtained NHS Health Research Authority Ethical Approval in June 2021 (21/EE/0104). The primary research question for the study, which will contextualise the methodological adjunct of Longitudinal Experiential Concepts to LIPA below, was *how do women experience anxiety during the perinatal period?* Participants were identified via the antenatal clinic of a large teaching hospital, with clinic lists screened for potential participants prior to an approach by the lead author. Purposive sampling was used to identify women experiencing anxiety based on the Generalised Anxiety Disorder‐II scale. Data were sourced via semi‐structured interviews conducted over a 14‐month period between November 2021 and February 2023, with five women of mixed parity and ethnicity interviewed once prior to birth (antenatal) and twice within the first year post‐birth (early postnatal and late postnatal). This resulted in 15 datasets for analysis, which are considered an appropriate number for this method (Smith et al. 2021).

Analysis began on completion of the antenatal set of interviews, taking the ‘themes by time point’ approach (Farr and Nizza 2019). The methodological steps described above were followed, with the principles of moving backwards and forwards between the themes, interview excerpts and reflexive annotations a key part of this process. Nine Group Experiential Themes emerged, and the novel addition of three Longitudinal Experiential Concepts illuminated a holistic view of lived perinatal anxiety. The condition was revealed as a collision between the social constructs of anxiety and motherhood, with existential notions including relationships with self, others, and the world central, as reported elsewhere (Folliard et al. 2024).

**FIGURE 2:**
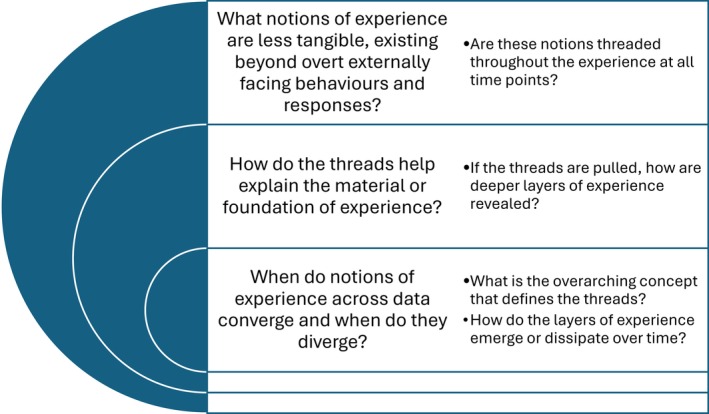
Reflexive prompts to assist with formulation of Longitudinal Experiential Concepts in Longitudinal Qualitative Research.

**FIGURE 3:**
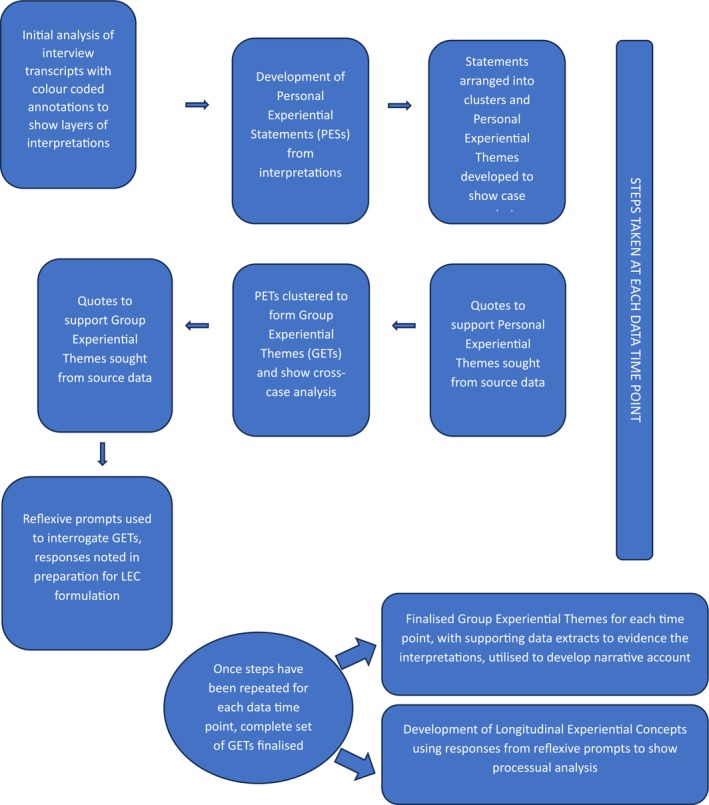
Stages of data analysis with Longitudinal Experiential Concepts formulated after assimilating the Group Experiential Themes, using responses from the reflexive prompts.

The use of iteratively derived reflexive annotations is a central element of LIPA used to formulate interpretations and enable data interrogation and thematic development, an example of which is seen in Figure 1. We suggest that additional deliberate use of reflexive prompts (Figure 2) is a step that can be purposefully added to the analytical process to enable the development of LECs by other researchers. The prompts can also be used to interrogate the LEC iteratively with cross‐checking between time points. Figure 3 shows that the LECs are formulated once the entire set of Group Experiential Themes has been assimilated, alongside the development of the narrative account, using previously noted responses to the reflexive prompts.

Below we provide detail on how incorporating LECs can address analytical issues and consider how this method can add interpretative and longitudinal integrity to a LIPA study.

## 4 Overview of the Issues

The pragmatic choice of analysis by time point was based on the need to gather, analyse and manage data over a prescribed course of time within the deadlines of part‐time academic study, and alongside part‐time clinical midwifery practice. There was no capacity within this for periods of time between the antenatal and postnatal interviews where analysis was not being progressed. The competing demands of part‐time academic and clinical practice often in tandem with caring responsibilities are a unique challenge for nurse and midwife researchers; professional registration and clinical careers are prioritised with research engagement occurring later compared to an exclusively research‐based career focus (Baldwin 2013; Smith 2018a). Even outside the contextual challenges for clinical nurse and midwife researchers, dealing with the volume and complexity of data collection and analysis is one of the challenges of longitudinal qualitative research and needs careful management (Neale 2021).

There was therefore a dual challenge in undertaking the perinatal anxiety study, with its planned data collection period of 14 months, to address logistical challenges while carefully managing the longitudinal qualitative approach. It was necessary for the analysis of time point 1 (antenatal) data to commence before time point 2 (early postnatal) data collection, and the analysis of time point 2 prior to data collection at time point 3. It was not practicable to take a ‘themes spanning time’ approach, which would mean waiting until the completion of the final data collection for each participant, before developing themes relevant to specific aspects of experience.

The themes by time‐point approach to analysis may therefore be the pragmatic choice for LIPA nurse and midwife researchers. However, the authors found in practice this raised questions about the ability to maintain an overarching (horizontal) experiential focus throughout the work and push the analysis beyond the gathering of potentially superficial time‐bound themes, which was important both for grasping temporal experiential perspectives and providing a view of the whole phenomenon. A sense arose early in analysis that something was missing that would contemporaneously capture, follow and interrogate the unfolding experience; this became most powerfully evident during the second phase of data collection. The missing piece was the need for a way of understanding the progression through the time points, for the narrative to remain cohesive and so that the ‘series of wholes’ (Farr and Nizza 2019, 200) that create the overall interpretation did not become disparate units. This is a common challenge in longitudinal qualitative research, favouring a cross‐sectional, lateral analysis over a deeper diachronic (across time) view, and losing the opportunity for potential analytical insights informed by the longitudinal approach (Neale 2021).

Introduction of the novel methodological step, Longitudinal Experiential Concepts (LECs), arose iteratively within the process and enabled a means to address these challenges. Initially considered by the authors as nuanced experiential temporal threads, these LECs were added to the methodological process and imagined as conceptual anchors, attended to after assimilating the entire set of Group Experiential Themes and alongside the development of the narrative analytical account (Figure 3).

## 5 Findings

The attribute of the ‘conceptual anchor’ was ascribed to the LECs as they emerged as a stable point of reference both within and across the timepoints from which interpretations were formulated. With analytical work overlapping and taking place over an extended period, keeping a grasp on all the cases and maintaining interpretative and analytical validity was a clear concern. The LECs provided a way of interrogating both the deeper meaning of the phenomenon and the validity of the interpretation over time. The need to maximise the longitudinal data for the value it would add to the understanding of the phenomenon was recognised:

How does the fact that the data is longitudinal add to the argument about the uniqueness of perinatal anxiety? Personal reflexive diary entry, August 2022

Neale (2021) notes that temporal and interpretative integrity in longitudinal qualitative research is maintained through the ways in which studies remain true to the changing worlds of the participants, following threads backwards and forwards through time and seeking to understand how processes reoccur. The LECs enabled a hold on these temporal threads across the different analytical time points while simultaneously focusing on the time‐point specific themes. The existential nature of the threads became apparent when reflecting on ideas about the uniqueness of perinatal anxiety and the participants' transition from pregnant women to mothers:

… anxiety takes on a new form. And that form is bound up in the ‘being’ pregnant, – it is about who women are in that moment: responsible, protecting, a vessel, worthy of being a mother, needing not to fail, fulfilling a primal function. Personal reflexive diary entry, August 2022

The threads which started to crystallise as LECs enabled a broader view of the phenomenon beyond the immediately evident behavioural responses to anxiety. The LECs as anchors facilitated a point of stillness within the ‘dance’ of the phenomenological attitude (Finlay 2011, 74) moving towards and away from the data to see the temporal experience in its entirety, enabling the whole experience to come into sharp relief as the focus becomes *just right*:

…when you are trying to get the focus just right and you zoom out or in too far and you can't see clearly – you have to get it just right for it to come into full focus…. Personal reflexive diary entry, May 2022

While providing an anchor within and between the time points, the LECs, as subtle and overarching characterisations of experience, simultaneously encouraged deeper more nuanced thinking that enabled the phenomenological attitude and emergence of philosophical notions. Highlighting the less apparent experiential concepts was informed by Gadamer's interpretative horizons, seeking greater depth of understanding by searching for what lies beyond superficial or immediately evident facets of experience. Gadamer (2013, 246) described the ‘unified flow of experience’ formed by implicit horizons joining with the continuum of experience, within which horizons do not limit interpretations but provide a means to greater understanding (Vessey 2009).

This notion connected to the sense that had arisen during analysis that there was something that lay beyond and was not fully captured through the Group Experiential Themes, something less tangible than superficial observed behaviours and observations, but key to the holistic experience of perinatal anxiety. The interpretative process revealed this core of experience present for the participants at each time point and throughout the continuum, illuminating broader conceptual layers:

It's like there are these layers on top – what we outwardly see in behaviours, what we can relate that to superficially – but then it feels like there is a layer underneath which is about existence and life. Personal reflexive diary entry, August 2022

Chiming with Husserl's life‐world phenomenology (Gadamer 2013), parallels of significance in clinical practice are evident in the work of Taschereau‐Dumouchel et al. (2022). This work advocates for practice that challenges prioritisation of objective (behavioural and physiological) measures of mental state over subjective measures, noting the risk of failing to identify subjective suffering, which if understood provides valuable clues to the potential for appropriate treatment. This supports clinical impetus for the holistic view of experience, which the LECs sought to reflect through augmenting understanding of subjective conscious and non‐conscious experience. Thoughts on these deeper layers of meaning supported theoretical ideas regarding the uniqueness of perinatal anxiety as a distinct condition, the overt and hidden aspects of the experience and the exploration of existential notions which followed:

The thing that is unique is about the person you are at that moment, not the way the anxiety manifests and the psychological themes (behaviours) you can draw from that. Personal reflexive diary entry, August 2022

**FIGURE 4:**
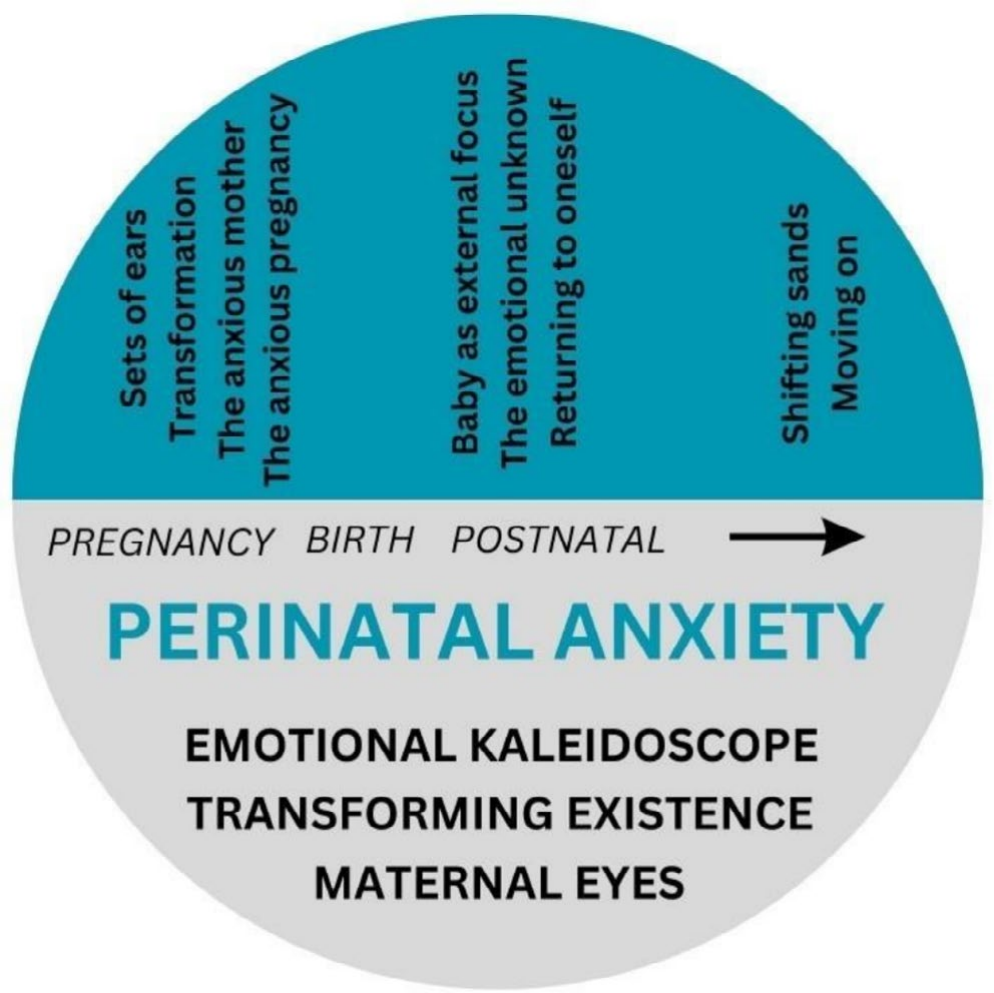
The lived experience of anxiety over the perinatal continuum showing the Longitudinal Experiential Concepts as the bedrock of experience (Folliard et al. 2024).

By pulling experiential threads, the material of perinatal anxiety is revealed and the formation of LECs enabled greater understanding of the flow and central characteristics of experience within the phenomenon and over time. This facilitated a holistic model for the lived experience of perinatal anxiety, with the LECs the less immediately obvious but fundamental bedrock upon which conscious experience is formed, represented across and supporting the Group Experiential Themes throughout the continuum (Figure 4) (Folliard et al. 2024).

The three LECs, entitled Maternal Eyes, Transforming Existence and Emotional Kaleidoscope (Table 1), arose iteratively and spread across all time points with a purpose that was twofold. First, they reflected experiential facets that were nuanced and phenomenological, enabling the idiographic detail that is a feature of IPA analysis (Smith et al. 2021). Secondly, they facilitated the maintenance of a thread across the temporal evolution of experience that acted as an anchor through the longitudinal analysis. This provided a reminder of what had previously emerged, allowing interrogation of the ways in which experience evolved, and the past was connected to the present.

**TABLE 1:** Longitudinal Experiential Concepts with Group Experiential Themes and subthemes by time point (Folliard et al. 2024).

Maternal eyes (LEC)	Transforming existence (LEC)	Emotional kaleidoscope (LEC)
**The anxious mother (AN)**	**Transformation (AN)**	**Sets of ears (AN)**
Otherness	Fighting with self	Feeling heard
Burdens	Temporal collisions	Safety net
	Reflecting and self‐understanding	
**Baby as external focus (EPN)**	**Returning to oneself (EPN)**	**The anxious pregnancy (AN)**
Distraction	Finding the way back	Lonely and unmagical
New worries	Looking forward	Grasping psychological safety
**Omnipresent (LPN)**	**Moving on (LPN)**	**The emotional unknown (EPN)**
	Coping	Relief and overwhelm
	Acceptance	Comfort
	Resolutions	Optimism
		State of flux
		**Shifting sands (LPN)**
		This too shall pass

Abbreviations: AN, antenatal; EPN, early postnatal; LPN, late postnatal.

### 5.1 Maternal Eyes

The Maternal Eyes LEC was seen across the GETs *the anxious mother* (antenatal) and *baby as external focus* (early postnatal) and in the subtheme *omnipresent* (late postnatal). It represented the critical significance of felt maternal responsibility and notions of relationships with self, others and society, how the participants see and are seen. To demonstrate the identification of this thread in the data, some examples underscoring Maternal Eyes woven throughout the antenatal, early postnatal and late postnatal GETs, are shown with the related expressions in bold (Table 2). Maternal responsibility and the temporal perspectives of the participants as pregnant individuals and mothers were revealed as central to the psychological adaptation that distinguishes this type of anxiety. Temporal evolution was seen as the uniqueness of the maternal view remained a key feature of the anxiety experience beyond pregnancy and into the months following birth, with maternal responsibility and relational aspects consistent.

**TABLE 2:** Expressions supporting the Longitudinal Experiential Concept ‘Maternal Eyes’.

**Maternal Eyes Longitudinal Experiential Concept—example expressions** (AN, antenatal; EPN, early postnatal; LPN, late postnatal)	
Maternal responsibility and relationships with self, others and society	‘Yep so um there have been times where… I have thought to myself, erm, probably **I shouldn't be carrying now**’ (Meena, AN)
	‘I've read that you know that **mothers with anxiety** or depression or mental health issues, er… (their) **children can develop problems** with, you know, in their life like ADHD and stuff like that yeah so you know it certainly has er, erm **worried** me. It still **worries** me, I'm not gonna lie’ (Meena, AN)
	‘When I'm pregnant it's **me and only me that can protect** her. So yeah, I think being pregnant makes it a little bit worse because **everything I'm doing and everything of how I feel I just didn't want it to impact her**. I don't want her to come out and be a worried baby, I want her to come out and be a happy baby, do you know what I mean?’ (Gabi, AN)
	‘You want to do everything **best you can to protect** it and make sure everything is going ok, best you can before she's born. Make sure everything is ready and you keep on top of things. It's like wrapping someone up in cotton wool. I know they say you shouldn't do it but it is something that it does definitely’ (Kate, AN)
	‘How do I explain to a four year old that the baby died? I just lay there replaying the conversation in my head that I'd have to have and feeling like **I'd failed her as a mum**, because I didn't fight hard enough to get the support I needed…(tearful)… even though I was doing everything I physically could. And you know and having to see my husband lose a child and my in‐laws lose a grandchild, it just felt like it was **all on me** to keep this pregnancy and this baby alive’ (Sam, AN)
	‘I think **a lot of women do put it out there** that it's the most amazing, magical time and every day you should be grateful and not complain about how you're feeling. I think when you've had a really difficult experience where the pregnancy isn't that easy, it **should be a little bit more normalised** I guess that it's really OK to not enjoy it’ (Lucy, AN)
	‘I could **look as normal** as they come and yet I have this every day’ (Gabi, AN)
	‘We got him here, he's now my baby, he wasn't my baby that was at risk of stillbirth or at risk of premature labour or any of the complications with obstetric cholestasis. He had jaundice but I expected that because my daughter had jaundice. But everything else was in my control, **he was my baby and nobody could tell me anything else**. He was the other side of my stomach’ (Sam, EPN)
	‘I just ended up being, not that I didn't want to be involved with him, I just couldn't, **my whole feelings and stuff had shut off. I think out of fear of hurting him**. I just didn't want to be in the same room as him, didn't want to go near him’ (Lucy, EPN)
	‘God it has been horrible. Anxiety in itself, then **when you put a baby into the mix, it's like something you can't fully describe without feeling it**, the amount of stuff that runs through your head, it's all the things that are negative about what could go wrong’ (Lucy, EPN)
	‘Surreal is the word. Erm, I know I had a baby but it's amazing to see her out. And you know like I said when I first saw her, even though, you know, you had a baby, you always wonder how she would be and everything. It's not… it feels like magic or something you know you can actually see her out and how she adapts. And, you know, **she knows you're mum already** and she wants to be with you and have a cuddle and everything straight away’ (Meena, EPN)
	‘Having Mia here I'm obviously **not as lonely** anymore, there is **another person**’ (Kate, EPN)
	‘I would say it has definitely improved. Yeah. There are **still little bits that need a little bit of work** on and are a bit rough around the edges but all in all I would say yeah, I am a lot better’ (Gabi, EPN)
	‘I had anxiety with leaving the house, going out, that side of things, which that has somewhat got a bit better to be honest. Because I feel like I'm not going out the door on my own and worrying about me, I'm going out the door and worrying about her. So **it's taking my mind off me**, which is making my own anxiety better in a way but **the anxiety has moved to her**’ (Gabi, EPN)

	‘…that's the only way I can describe it, **people didn't treat me like a mum** so I didn't feel like I was a mum’ (Sam, EPN)
	‘Yeah, and I was anxious about… **if people knew what they would then think of me**, whether he was going to be taken from me, and then every bad scenario came into my head. I told my partner not to tell his mum what was going on because I just didn't want to be **judged or branded** like a bad mum from something that I had no control over’ (Lucy, EPN)
	‘Yeah, you know where she is to **keep her safe**, and you're doing what you're doing to **keep her safe** [in pregnancy]. Whereas obviously she's out now and you're paranoid, well I'm paranoid, of who touches her… So it's just constant now because she's here and she's in front of you… it's like **if she's here I can see she's ok**. But at the same time I am a little bit more fearful because she is completely there ‐ I can't just rub my belly and know she's alright, I have to **keep an eye on her** all the time’ (Gabi, LPN)
	‘But my, what's the word… **I wasn't worried about me as a person but I was worried about me as a mother**. I had another child, so I was having to survive for that child while trying to **make sure that this child survived**’ (Sam, LPN)
	‘Every day I think I don't want to go out I don't want to do this. I've got a friend and I'm at no. 24 and she's at no. 14 and **I haven't seen her for a month** and a half. So yeah I have been quite **bad with not going out**, I think it's either too hot for her and I don't want to get her burnt and even in the shade it's too hot. And now it is raining, So what do you do?!’ (Gabi, LPN)
	‘So after we finished the baby massage we went on to sensory classes. Things seemed to be getting better and then I started getting all of those bad thoughts and feelings and sort of **distancing again away from Charlie** and I **didn't really tell anyone** because I didn't want there to be a whole thing about it’ (Lucy, LPN)
‘But since having the baby it has been a lot lot better, even though like I say I have good days and bad days, when I do have bad days I have this new kind of plan or technique where I say the baby's name 10 times and look at her and **concentrate on her instead of the source of the anxiety**’ (Meena, LPN)

### 5.2 Transforming Existence

The presence of existential themes within the data was striking, and although the participants did not use the language of philosophical dimensions, their concern with existential notions which gave rise to the LEC Transforming Existence was evident across the GETs *transformation*, *returning to oneself*, and *moving on*. Related expressions for this LEC are shown in Table 3. This LEC illuminated expressions of identity, temporality, mortality, and the unknown. These concerns were again woven through the data across the whole experiential research timeframe, verifying the relevance of existential notions within the complete experience of perinatal anxiety. Temporal perspectives were seen in the cognitions, behaviours and meaning the study participants derived from experiencing perinatal anxiety as they formed future and past projections, where in common with ‘Maternal eyes’ and ‘Transforming existence’ temporal perspectives of themselves as individuals and mothers are central to psychological adaptation.

**TABLE 3:** Expressions supporting the Longitudinal Experiential Concept ‘Transforming Existence’.

**Transforming Existence Longitudinal Experiential Concept—example expressions** (AN, antenatal; EPN, early postnatal; LPN, late postnatal)	
Identity, temporality, mortality, and the unknown	‘So I think you're just **fighting with yourself** trying to do things because you don't know if you'll experience it again but at the same time your head is just shutdown. It's like you can't do this, you just need to stay in the house. Something will happen if you go out and it's just quite difficult I guess’ (Lucy, AN)
	‘… through the pregnancy I then notice I try not to be so anxious where, **if I wasn't pregnant** and I had an appointment **I'd think balls to that and I'd cancel it**, I'd just think I can't physically do it today’ (Gabi, AN)
	‘I used to love just getting up and going out and **I was definitely more spontaneous** than I am now and I would just be let's go here, let's go there, let's go out and enjoy the day. Whereas now it has to be planned and if I have a bit of an off feeling about it, it won't happen… **I suddenly switched one day**’ (Lucy, AN)
	‘Due to the anxiety I had, I had suicidal tendencies as well, erm, **that part of me my worst, worst ever during my pregnancy** but erm ever since then I still have had quite a few lows’ (Meena, AN)
	‘I **felt like I was going crazy**. I started to believe them, like maybe I'm not, **maybe this is all in my head**, maybe I am just being a hypochondriac and maybe… and then you think I can't think like that because if I am right I'm putting my child's life at risk… I was laying awake for hours playing through the conversation, questioning myself, like am I actually going insane right now? And is what everyone is telling me right, or **is what I know I'm feeling, what I know is right**?’ (Sam, AN)
	‘And then you're like thinking **when I'm not pregnant** am I going to **look back** and wish that I had done more? (Lucy, AN)
	‘My aim of the game is to **concentrate on my future**. To concentrate on getting out of the house, letting my kids live a little, taking them to places they want to go without me worrying’ (Gabi, AN)
	‘…the best that it can be **as far as I've ever felt by my experience**. Because my experience during pregnancy, it was one of the lowest’ (Meena, EPN)
	‘You know, that helps me out as well because it gives me a reminder when I do start thinking well actually **I've got a right to be here** as much as anybody else, I'm just being stupid, you know I change the subject in my own head’ (Gabi, EPN)
	‘So having a routine has helped my anxiety. Not having a routine does not help my anxiety at all. So **knowing what I'm doing and when I'm doing it** is easier for me. So most of the time we have a routine where by lunchtime she has fed and is ready to go down for a sleep so I can then do what I want to do. So yeah not having a routine, that was hard, that was hard going. Because you just **didn't know what to expect and what to expect when** because each day was different…’ (Kate, EPN)
	‘Well [my partner] was there at most appointments and he was saying to the consultant she is literally **a shell of a person**, borderline hitting depression ‐ and that didn't make any difference. But yeah he said that he thinks I'm still not fully back to normal, but he knows I've got this worry in my head about becoming pregnant, but I know how I feel in myself and apart from that **I feel like me again**’ (Sam, EPN)
	‘I can't put myself mentally through it again. I said the same after the last one, and I can't do it again, **I have to be here** for the two children that I've got. And it wasn't like it was a one off, it happened both times’ (Sam, EPN)
	‘I also know that a lot of it is in my head and **because I know it is in my head I try and rise above it**. But other times it will come into force more and I will think I'm just not doing that’ (Gabi, LPN)
	‘It's a great change don't get me wrong, it's nice to know she can move around and do things. But on the flipside, it's not good because obviously **I don't get time to myself**’ (Kate, LPN)
	‘Just simply asking for a bit of help, can you feed him you know? Once a day can you make the bottle, can you put the washing on? Just simple things to **give you 10 or 20 min to make yourself some food** or make the bed, it really does make a difference’ (Lucy, LPN)

	‘Yeah, I think now it makes a lot more sense to me; I've looked into it a lot more and done a lot of my own research around it. And I've spoken more with the perinatal team and with doctors and things and it definitely is sort of like reassuring that a lot of women do go through it. It's a bit **reassuring that it's not just me** and it's not something I'm intentionally doing. You know hurting him or wanting to run away from him, it's just a chemical imbalance that I can't control. And I just needed some help with that’ (Lucy, LPN)
	‘It's definitely better, if I had to rate it now I would say it is less than five to be honest. It's quite an improvement, and I understand it can never be ‐zero ‐ that is just how life is. It's brought me to a stage or **a phase where I can go on with my day‐to‐day life** easily’ (Meena, LPN)
	‘Umm…basically not to worry. I think a lot of my thing is I was just paranoid, and I was a thinker, and when you think you overthink – when you overthink you go to the extreme. I think, personally, just don't think into it too much. Everything will be fine and **your body usually lets you know if it's not fine** so you know, **listen to your body as well**’ (Gabi, LPN)
	‘I think I'm a little bit more rational about things, I do have things that pop up in my head and stuff and I think **I can't think about that right now it's just not reality**. I can only think about **the moment and the next couple of months** at the max’ (Lucy, LPN)
	‘The only way I can describe it is that **now I am me and before I was just looking at me**. I felt like I wasn't there, **I was just seeing myself disappear**’ (Sam, LPN)
‘… weird because the feelings weren't what I was feeling about Charlie but **more towards myself**… I'd think God I've got such an easy baby but I'm still having all of these horrible thoughts and things towards him. What's wrong? You know. **I thought I was going crazy**’ (Lucy, LPN)

### 5.3 Emotional Kaleidoscope

The GETs *sets of ears*, *the anxious pregnancy*, *the emotional unknown* and *shifting sands* were framed by the LEC Emotional Kaleidoscope, for which example expressions can be seen in Table 4. The data demonstrated that the participants' experiences of perinatal anxiety were of multiple emotional dimensions, with notions of emotional lability, unpredictability and variance of the feelings evoked being central. The link between perinatal anxiety and guilt supported a phenomenological understanding of maternal guilt, with anxiety and fear as the precursors to this additional complex emotion (Pierce et al. 2022) and emotional wellbeing related to the presence or absence of support structures. This LEC built on the conceptualisation of perinatal anxiety as a continuum, a perspective missing from the theoretical discussion of perinatal emotional wellbeing (Wadephul et al. 2020), and was evident in the ways the participants reflected on their understanding of temporal perspectives.

**TABLE 4:** Expressions supporting the Longitudinal Experiential Concept ‘Emotional Kaleidoscope’.

**Emotional Kaleidoscope Longitudinal Experiential Concept—example expressions** (AN, antenatal; EPN, early postnatal; LPN, late postnatal)	
Multiple emotional dimensions: guilt, fear, lability, unpredictability, variance, support	‘Probably end of August/beginning of September, was when like I started being able to eat a little bit better. But again that's when the specialist midwives got involved so that took some of the stress off me because **somebody started listening**’ (Sam, AN)
	‘My **partner could have been a bit more supportive** – that's the only thing I would say would have changed it dramatically, I wouldn't have gone into such a low point otherwise’ (Meena, AN)
	‘I felt there was a lack of support and **I felt kind of like I was doing it all by myself**. Although there was like my partner, but like you know **outside of the home there wasn't anybody**’ (Kate, AN)
	‘I just felt like, obviously I know the child is always going to mean more to us than anybody else, but I just **felt like no‐one even cared. I was just another number**’ (Sam, AN)
	‘So it's either like erm I'm **expressing my anger or I am being completely like a wall**, I've got a wall around me’ (Meena, AN)
	‘That's when it hit me that oh my god, I'm carrying this child and I don't know what I'm doing, this is my first time, **all these different feelings going through my head and in my body** and it just kind of just set me off on like a kind of a low, like er, what do you call… a low cycle’ (Kate, AN)
	‘The only way I can describe it is **I was numb**… Because, because I had no fight left in me… I wasn't eating, I was just literally laying on the sofa, I **didn't get dressed for days, didn't function**’ (Sam, AN)
	‘We paid a silly amount of money so that every month we could have a scan and I was **constantly Googling whether my symptoms were right**’ (Lucy, AN)
	‘During pregnancy I was **terrified**, I was **terrified** for how things were going to be when I had Nell. If it would be, with the way things were in my pregnancy, way way worse having Nell around’ (Meena, EPN)
	‘You feel them wriggling around and you just assume they're okay, but **you don't really know until they come out**. There's only so much a scan can show and so until they come out **you don't really know**’ (Kate, EPN)
	‘It was different the moment he was put on my chest. **It was just relief**. I didn't even realise how much pain I was in with my placenta for like half an hour… I just remember crying as soon as he was on me, **it wasn't happiness it was just pure relief** that it was over and that this is a bit that I know I can do, and no one else has an opinion because he's my child now’ (Sam, EPN)
	‘I looked over and was **panicking** because I was watching for her to breathe but she was in such a deep sleep that her breathing was really shallow… I'm **constantly up checking her**, even though she's not making a noise, she's just sleeping but I'm **constantly up checking**’ (Gabi, EPN)
	‘…it **puts me on edge**, because I don't know what to do. And my partner, he doesn't like it when she's crying so much and just wants to soothe her, but he can also get quite stressed out with her crying. There's me trying to stay calm and then he's not getting cross with her, he's getting cross with himself. Which then in a way sets me off in the fact that **I don't know what to do, what else can I do**?’ (Kate, EPN)

	‘… it wasn't that I just didn't want to be around him and didn't want to be involved, it was that I physically couldn't. **The emotion just wasn't there**’ (Lucy, EPN)
	‘I would say that even though it's **horrible to be switched on and aware** of people around, it's tiring like constantly watching other people and making sure he's alright, but it's **also quite reassuring** that I have that worry about him, if that makes sense? Instead of like being Oh yeah he's alright, someone can wander off with him and I'm not very bothered… I think I would prefer that over not having any worries towards him’ (Lucy, EPN)
	‘I'm less anxious and it's **more joyful**. It's just all going well, touch wood, and hopefully it stays like that. The only difference is that **the anxiety was from one extreme to the other**, it was worse during my pregnancy. It's nowhere near perfect, but it's definitely better’ (Meena, EPN)
	‘…sometimes now I'll go out like to my mums or something, and I won't have to take any lorazepam and yeah if my partner has a really busy day and I just feel like popping out or something I just get Charlie ready and we just pop out for a coffee or something, or go round friends and **sometimes it's easier than others**. I don't know, I just wake up **a little bit more anxious some days than others**, so I'll take a lorazapam or something…’ (Lucy, EPN)
	‘I would probably say ‘this too shall pass’. Because I think **looking back to me it felt is this it, is this is the end** ‐ but it's not, **it's just an emotion which comes and goes**. I have good days and bad days, and so that is what I would tell myself’ (Meena, LPN)
	‘…when my anxiety kicks in, **I'm normally on my own and then I panic**. Whereas when I am with her I find my anxiety kicks in then I can distract myself by playing with her’ (Gabi, LPN)
	‘I keep telling myself what happens in the future happens in the future, **I need to live in the here and now**. Some things you can plan ahead and other things you can't, some things you can't plan at all. So yeah, we just sit in the here and now and **when the future comes the future comes**’ (Kate, LPN)
	‘Um… so every ounce of mental energy I had was fighting to get me through this pregnancy to make sure I was going back to the child I had. Because **I couldn't love him yet because of the risk of him not being here**, and I didn't know him, not like I know my daughter’ (Sam, LPN)
‘I think over the time that we've been talking it's weird to see the differences in how anxiety can change. Like even though **it seems a long period of time realistically it's actually a short period of time**. And I think going from the anxiety that I had before pregnancy and then during pregnancy and after pregnancy it's just really **weird to see how it can change so quickly**. I guess it can even change daily, my anxieties and what's going on and everything’ (Lucy, LPN)

These examples demonstrate how the LECs as conceptual anchors can begin to be identified once the GETs are finalised, with further synthesis while writing the narrative account (Figure 3). They are underpinned by supporting statements within the original data and cross‐referenced to the data sets for each time point to confirm they are present throughout the whole temporal process (Tables 2, 3, 4).

### 5.4 Formulation of LECs and Use of Reflexive Prompts

**FIGURE 5:**
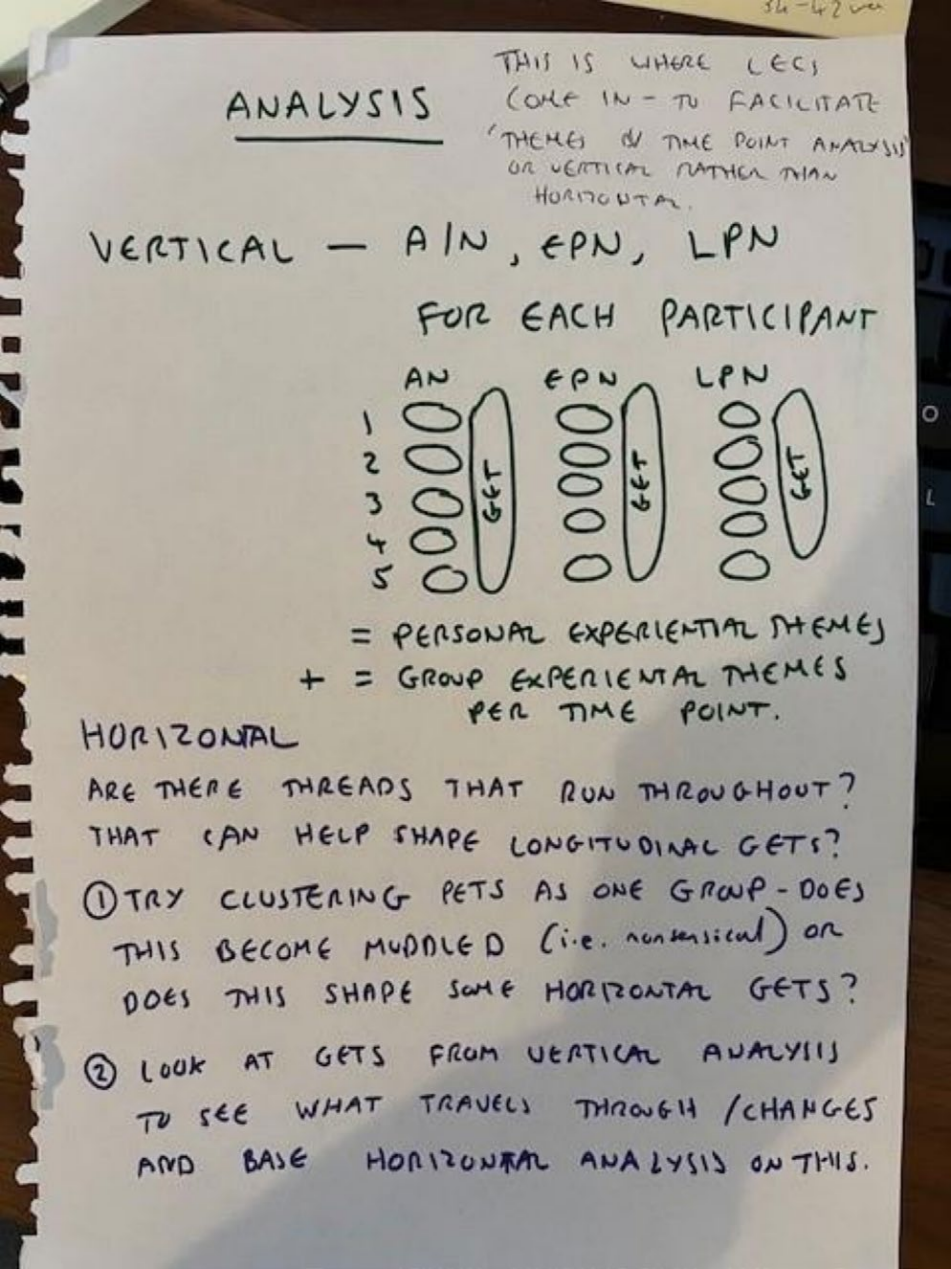
Researcher annotations—understanding how to address the temporal view while dealing with vertical themes by time point analysis.

We sought to maintain the longitudinal integrity emphasised by Neale (2021) through the LECs, formulation of which began following assimilation of the entire set of Group Experiential Themes (GETs). At this point the GETs risked being disparate entities tied to their point in time; the need for a means to develop a holistic temporal view demonstrating processual questioning and enabling the interpretation of change over time (Neale 2021), became apparent (Figure 5). The GETs were reflexively interrogated to ask which experiential threads had been present throughout the data, and which facets of experience were less immediately apparent, representation of which provided an overarching view of the phenomenon (points 7–8 Appendix S1).

**FIGURE 6:**
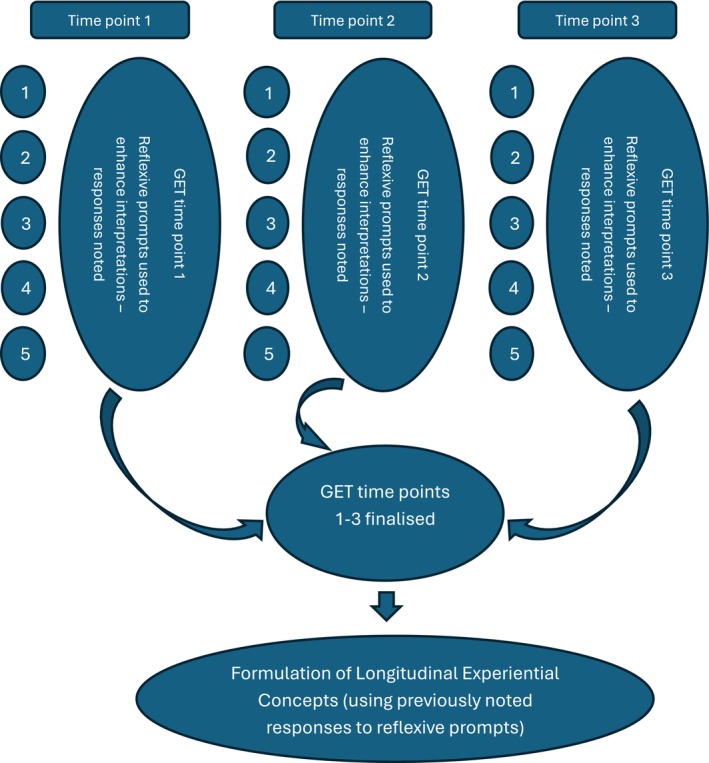
View of vertical data analysis, example with 15 datasets over 3 time points. Data set shown for each participant (1–5) at each time point (1–3) showing reflexive prompts used with the formulation of the GETs at each time point. The LECs are developed once the GETs for all time points are finalised, utilising previously noted researcher responses to reflexive prompts.

In this perinatal anxiety study the LECs were derived iteratively, with an example illustrating the process towards the development of the LEC ‘Transforming Existence’ provided (see Appendix S1). However, future use of the LECs as an additional methodological step within LIPA would be enabled by the deliberate use of reflexive prompts to guide this form of questioning more explicitly (Figure 2). The prompts can be used within the process of identifying the GETs for each individual time point (Figure 6). The researcher's responses to the prompts are held until the GETs for all three time points have been established, at which point the responses can be revisited to enable the development of the LECs.

The prompts are designed to guide and encourage the researcher to look beyond the immediately obvious facets of experience to what might be hidden, and to consider these obscured notions temporally as a form of processual questioning (Neale 2021). In this perinatal anxiety study, temporal interpretative validity for the LECs, with their focus on the view of the anxious mother looking upon her world (Maternal Eyes), the sense of existential transformation (Transforming Existence) and the emotional lability, unpredictability and variance of the experience (Emotional Kaleidoscope), is confirmed as each is underpinned by antenatal, early postnatal and late postnatal Group Experiential Themes and subthemes.

The LECs as conceptual anchors therefore added depth of understanding to perinatal anxiety and its progression over time, moving the condition beyond naturally objectively measurable perinatal‐focused anxiety behaviours, to a threatening phenomenon of far greater existential significance. Looking at connections across time and considering how to characterise these connections was where the vertical and the horizontal analytical views met, with the Longitudinal Experiential Concepts enabling the robustly holistic temporal perspective.

## 6 Discussion: Midwife and Nurse Researcher Perspectives and IPA

This paper has so far contextualised how Longitudinal Experiential Concepts were used as conceptual anchors within a perinatal anxiety study, detailing the rationale, development and use of this approach. The value of this methodological adjunct in enabling a robust temporal and phenomenological view has been highlighted. Extending this discussion further through the reflexive lens of a midwife researcher new to this longitudinal qualitative phenomenological approach, and responding to some relevant critical challenges, aims to foster understanding and confidence in nurses and midwives exploring the method.

When planning the study methodology, the first author's reflexive diary noted confidence in the choice of IPA, with accessibility due to existing methodological guidance (Smith et al. 2021), and faith in its suitability to enable the research questions to be addressed:

I am feeling really comfortable with the IPA choice, and so far everything I have read about it only confirms it as the right choice for doing the kind of exploration of experience that I am aiming for. Personal reflexive diary entry, October 2020

Moving from this naïve awareness around phenomenology as a qualitative research method to the sharpened focus required once undertaking longitudinal data collection and analysis, brought a sense of the complexity within the chosen approach. The potential fragmentation of the process began to feel like a pressing issue:

… this process feels so fragmented. Trying to get stuck into analysis when you are grabbing snippets of time is really challenging… even more complicated with this longitudinal design. Personal reflexive diary entry, May 2022

IPA is a methodological approach suited to phenomenological nurse researchers due to enabling focus on the patient voice in an accessible and adaptable manner (Pringle et al. 2011) and the potential for exploration of clinical decision making (Anderson 2019). The tension within IPA as a straightforward and paradoxically complex process is noted by Biggerstaff and Thompson (2008), who also point to the need for accessibility among IPA nurse researchers who are motivation‐rich and time‐poor, fitting postgraduate research alongside busy clinical practice, which was the experience of the lead author. Pereira (2012) describes the uncertainties felt by doctoral nurse researchers approaching phenomenological methods concerning how to robustly apply philosophical principles, an issue compounded by the diverse and at times contradictory nature of published opinion and guidance.

Interpretative Phenomenological Analysis has inspired critique and debate, including on its use by doctoral researchers. Paley (2016) considered the methodological distinctiveness of Phenomenological Qualitative Research as a whole, noting that methods using phenomenology, grounded theory and narrative enquiry have evolved to suit postgraduate research. Paley argues this has made it hard to distinguish between these hybrid methods which cross between meaning attribution, causal hypotheses and common themes. He argues that driven by publication productivity methodologists have superficially adopted philosophical ideas to justify a theoretical basis, which if experience‐centric negates the need to consider theory more broadly. For nurse and midwife researchers to effectively use IPA and robustly generate new knowledge, they must respond to the potential challenges of the method. This means having access to guidance, provided via accounts which specifically address measures taken to counter the critique described.

Giorgi (2010) also noted that when research methods are based on the philosophical underpinnings of phenomenology methodological variations can appear and a lack of understanding of phenomenology as a science compared to phenomenology as a philosophy can result in poor scientific application alongside inconsistency with the phenomenological view. Giorgi (2017) has however been sympathetic to naturally imperfect attempts to practice phenomenological science, for example by nurse researchers, who in most cases are unlikely to have solid theoretical training in phenomenological philosophy, and are further likely to be challenged by the developmental nature of this new science. Giorgi notes that the researcher adopting the phenomenological attitude to derive meaning and being explicit in their method is crucial to this approach (Giorgi 2017, 2010). This reinforces the importance of highlighting ways in which the phenomenological view can be enabled and consistency within the method aided, especially among novice researchers.

Larkin et al. (2006) argue that students using IPA must focus on the quality of the interpretative work to avoid completing a purely descriptive exercise that doesn't engage with phenomenological theory, and that poor IPA is easy to achieve while high‐quality IPA is challenging. Hefferon and Gil‐Rodriguez (2011) also note a tendency in IPA for heavily descriptive pieces of work, suggesting the methodology can be misunderstood as a form of thematic analysis, which results in low‐quality IPA. Lopez and Willis (2004) advise nurse researchers to choose a methodological approach that will substantially meet the aims of the research question in a meaningful way to further knowledge about the phenomenon under enquiry.

IPA aims to be translational in its coherent and pragmatic efforts to build knowledge of phenomena, via analysis of third‐person data based on the insights of phenomenological philosophy, the understanding of which may illuminate the embodied, cognitive affective and existential domains of psychology (Smith et al. 2021). It is essential for midwife and nurse researchers to uphold methodological and philosophical integrity to maintain the value of the phenomenological method in nursing science (McNamara 2005). The desire to address these types of concerns drove the methodological approach of this perinatal anxiety study. Dissemination of the process to enable future use of LECs as a novel adjunct aims to assist nurse and midwife researchers in responding to the critique.

Smith (2018a, 2018b) stresses that for scientific transparency and validity researchers should be explicit about how their methodological choices have manifested and influenced their work; however, he notes that due to the diversity of phenomenological philosophy there is no single definitive form of phenomenology. Dowling (in Thomson et al. Dowling 2011) discusses the development of phenomenology as a methodological approach in midwifery research and notes that rather than being a subfield of phenomenological philosophy, phenomenological psychology is a branch of psychology that is guided by philosophy. As noted by Smith and Eatough (2019), different approaches to IPA have been taken to advance the method conceptually and methodologically over the past 20 years and the application and distinctiveness of IPA continue to evolve.

The strength of this study is that it provides an additional step in the interpretative process to guide the novice clinical researcher who may be grappling with the science and philosophy of Interpretative Phenomenological Analysis. The position of the first author, not as a philosophy or psychology academic but as a clinical academic midwife pursuing a scientific endeavour aiming for the generation of new knowledge is acknowledged. The attention to robustness within the perinatal anxiety study described here was focused on constructing a compelling, unfolding narrative; developing a vigorous experiential and/or existential account; close analytic reading of participants' words; and attending to convergence and divergence (Nizza et al. 2021); the method was explicit and the desire to achieve the phenomenological attitude was a firm commitment (Finlay 2011). The addition of Longitudinal Experiential Concepts provided two key benefits: access to deeper temporal insights and the phenomenological attitude and addressing these methodological concerns to uphold the quality and validity of the work. In terms of limitations the pragmatic approach of identifying a way of linking time points through deeper analysis of the experiences at these specific time points may risk imposing patterns on to the data. However, it must be acknowledged that LIPA is an evolving method and this approach while working well in this particular study requires replication in others to show its utility.

## 7 Methodological Implications

The findings described in this paper, arising from a novice experience of undertaking a LIPA study, have implications for future use of the method. Via the additional methodological step of identifying LECs, aided by reflexive prompts, and using these LECs as conceptual anchors, users (especially those inexperienced with the method) of LIPA may find a means to robustly access deeper temporal interpretations, phenomenological insights and longitudinal integrity. This is likely to be of greatest value to those undertaking vertical analysis of themes by time point.

## 8 Conclusion

In a study examining the lived experience of perinatal anxiety using Longitudinal Interpretative Phenomenological Analysis, Longitudinal Experiential Concepts were found to be a useful methodological adjunct to facilitate themes by time point analysis while maintaining a horizontal view. These conceptual anchors, developed with the aid of deliberate reflexive prompts, can provide a bridge between the vertical ‘themes by time point’ and the horizontal ‘themes spanning time’ approaches to analysis. This can prevent fragmentation of experience and enable a contemporaneous temporal analytical perspective on the experiential threads that are woven throughout, enhancing longitudinal validity.

The LECs as conceptual anchors were also valuable in encouraging deeper holistic thinking about the less immediately obvious facets of experience to illuminate the whole phenomenon. Parallels can be drawn between this holistic theoretical view and clinical perspectives in determining effective care and treatment. As well as attending to issues of experiential congruence over time, LECs may provide the novice LIPA researcher a means to respond to the critique levelled at IPA doctoral researchers, including enabling access to the phenomenological attitude. The principles described here may be applicable more broadly; testing in non‐perinatal health contexts and with other longitudinal qualitative approaches is recommended.

## Conflicts of Interest

The authors declare no conflicts of interest.

## Supporting information


**Appendix S1:** jan70355‐sup‐0001‐Supinfo1.docx.

## Data Availability

We confirm that data utilised in the submitted manuscript have been lawfully acquired in accordance with The Nagoya Protocol on Access to Genetic Resources and the Fair and Equitable Sharing of Benefits Arising from Their Utilization to the Convention on Biological Diversity. The study received NHS Health Research Authority ethical approval in June 2021 (21/EE/0104).
