# Supplementary material for: Conceptual Anchors in Longitudinal Qualitative Health Research: Using a Methodological Adjunct in Longitudinal Interpretative Phenomenological Analysis
Source: J Adv Nurs. 2025 Nov 4;82(8):8212–28. doi: 10.1111/jan.70355 (PMC13356294; doi:10.1111/jan.70355)

**Supplementary information.**

**Example of data analysis process leading to formulation of Longitudinal Experiential Concepts. Example shows initial antenatal data interpretation and the process of development towards the antenatal Group Experiential Theme ‘Transformation’. Same process repeated at each time point for all participants:**

1. Annotating interview transcripts to develop Personal Experiential Statements.
2. Clustering Personal Experiential Statements to formulate Personal Experiential Themes.
3. Linking proposed Personal Experiential Themes back to interview data to confirm supported by source data prior to finalising.
4. Synthesising Personal Experiential Themes into Group Experiential Themes.
5. Development of Group Experiential Themes using supporting quotes to validate interpretation.
6. Finalising Group Experiential Themes (showing origin Personal Experiential Themes), including different iterations of labels for themes.
7. Making sense of temporal process and how to interpret change over time using time point specific Group Experiential Themes.
8. Re-thinking the idea of ‘Longitudinal Group Themes’ as Longitudinal Experiential Concepts.
9. Consolidating use and value of Longitudinal Experiential Concepts in providing holistic view and enabling a model for perinatal anxiety.
10. Development of Personal Experiential Statements showing interpretative comments and reflexive notations with original participant data. Repeated for each participant at each time point.


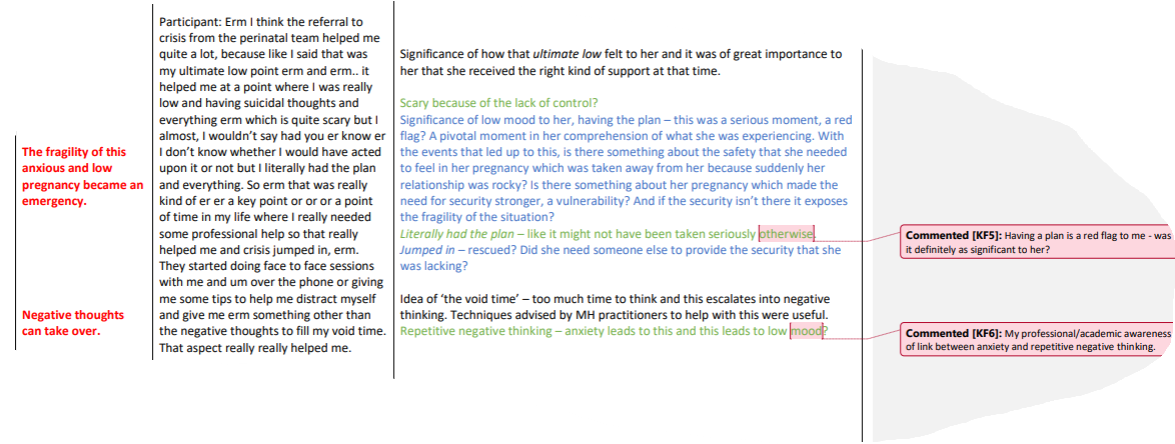


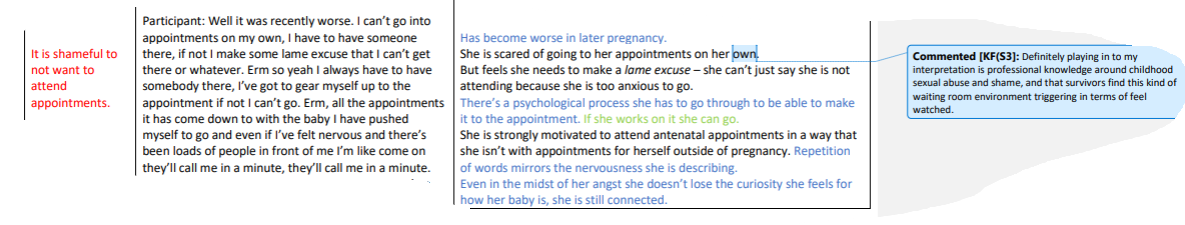


1. Personal Experiential Statements for each participant at each time point are clustered to enable development of Personal Experiential Themes.


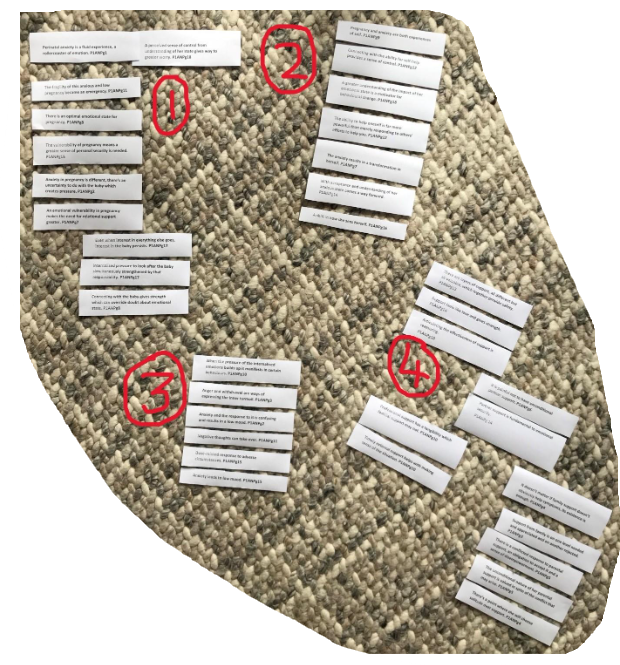


1. Finalising Personal Experiential Themes for each participant through mapping across of participant statements and quotes to evidence each theme.


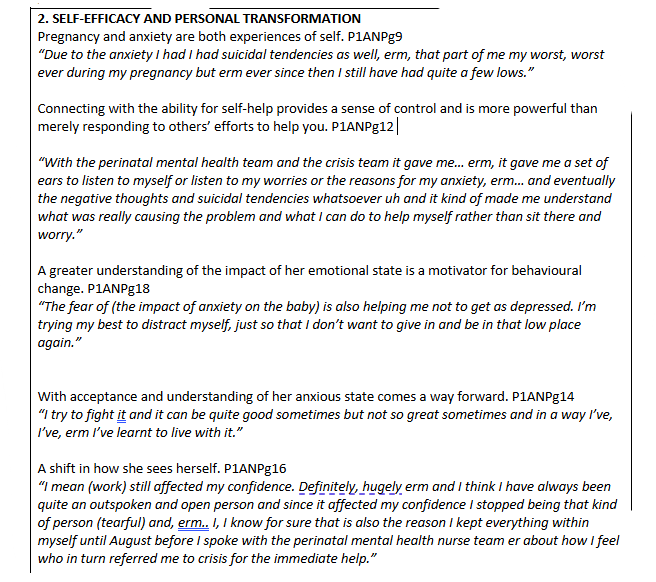


1. Synthesising Personal Experiential Themes into Group Experiential Themes with preliminary labels for antenatal Group Experiential Themes.


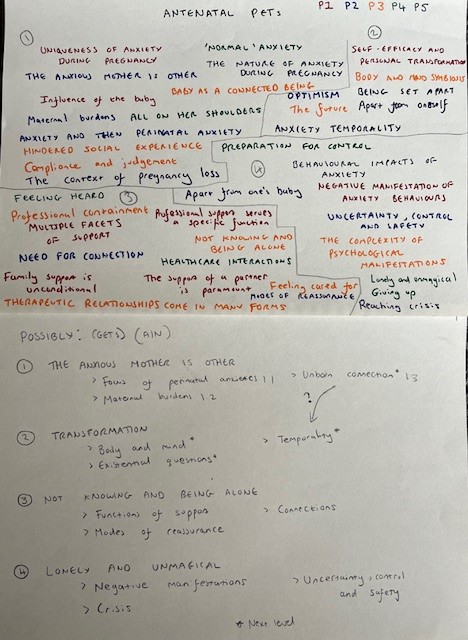


1. Validity of Group Experiential Theme (Transformation) evidenced by cross reference to quotes across all participants and showing reflexive notations.


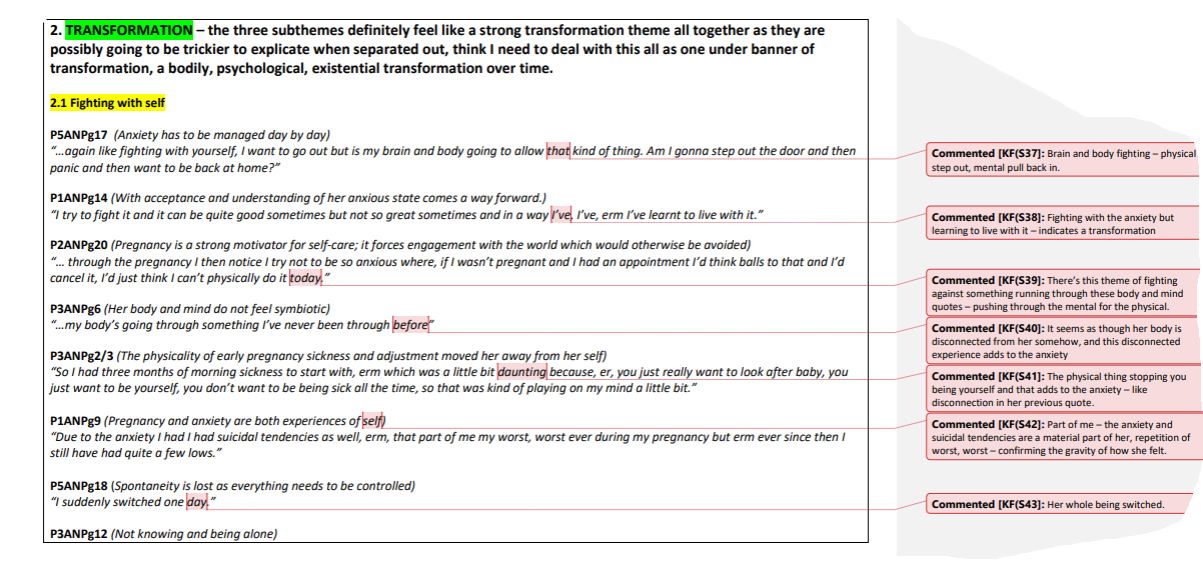


1. Development of Group Experiential Themes from Personal Experiential Themes showing emerging and finalised labels (highlighted and capitalised with subthemes underneath in finalised Group Experiential Theme column).


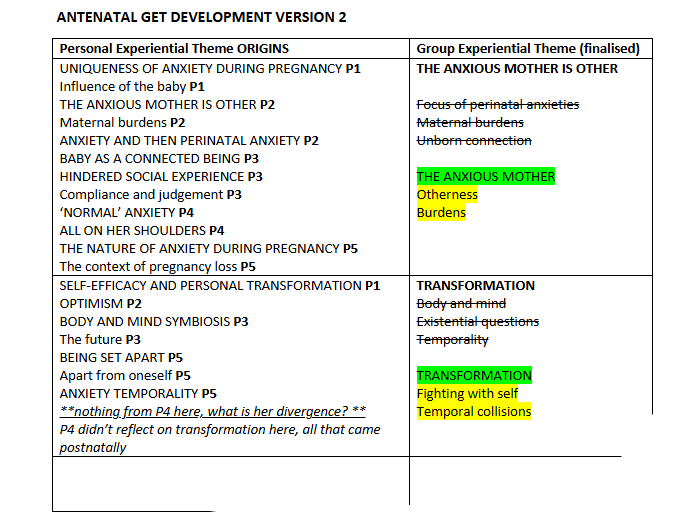


1. Making sense of temporal process and how to interpret change over time using time point specific Group Experiential Themes. Initial consideration of ‘Horizontal’ or ‘Longitudinal’ Group Experiential Themes’. Searching for a way of linking the whole experience together that accounted for temporal progression and the nuanced characteristics to find a holistic view of the phenomenon.


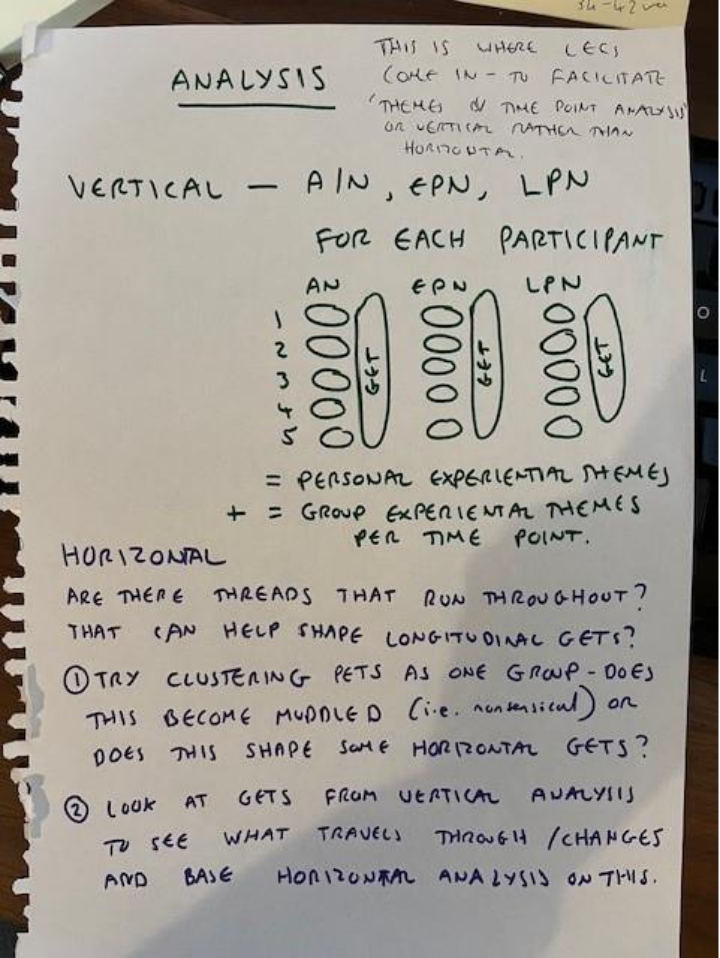


1. Re-thinking the idea of ‘Longitudinal Group Themes’ as Longitudinal Experiential Concepts. Beginning to understand how identifying Longitudinal Experiential Concepts could provide an overarching understanding of perinatal anxiety over time.


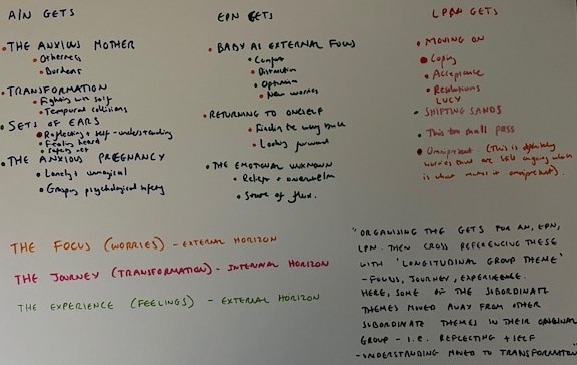


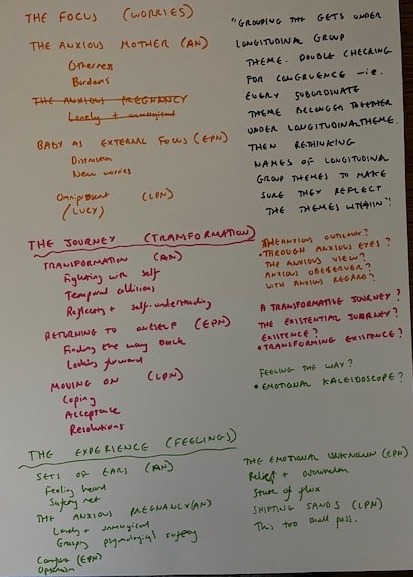


1. Consolidating use and value of Longitudinal Experiential Concepts in providing holistic view and enabling a model for perinatal anxiety.


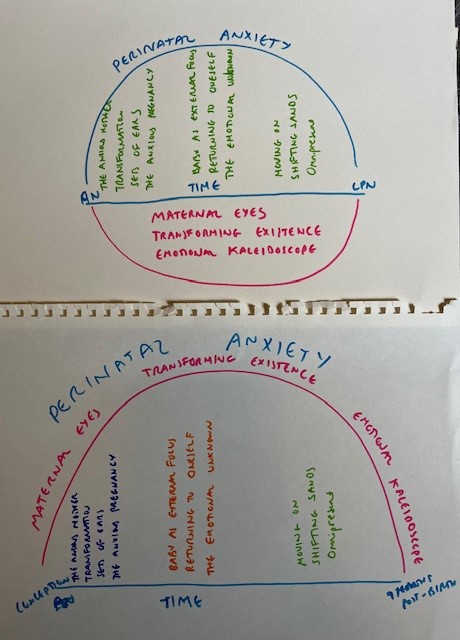

Supplement: Supplementary file 1 — Appendix S1: jan70355‐sup‐0001‐Supinfo1.docx. [file JAN-82-8212-s001.docx]
